# Weak nystagmus in the dark persists for months after acute unilateral vestibular loss

**DOI:** 10.3389/fneur.2023.1327735

**Published:** 2023-12-15

**Authors:** Chih-Chung Chen, Anand K. Bery, Tzu-Pu Chang

**Affiliations:** ^1^Dizziness and Balance Disorder Center, Taipei Medical University–Shuang Ho Hospital, New Taipei City, Taiwan; ^2^Taipei Neuroscience Institute, Taipei Medical University, New Taipei City, Taiwan; ^3^Department of Neurology, School of Medicine, College of Medicine, Taipei Medical University, Taipei, Taiwan; ^4^Division of Neuro-Visual and Vestibular Disorders, Department of Neurology, The Johns Hopkins Hospital, Baltimore, MD, United States; ^5^Department of Neurology/Neuro-medical Scientific Center, Taichung Tzu Chi Hospital, Buddhist Tzu Chi Medical Foundation, Taichung, Taiwan; ^6^Department of Neurology, School of Medicine, Tzu Chi University, Hualien, Taiwan

**Keywords:** nystagmus, vestibular, video-oculography, vertigo, dizziness

## Abstract

**Background:**

Weak nystagmus with fixation removed can be seen both in normal individuals and in recovery from a unilateral vestibular insult, thus its clinical significance is unclear in patients with dizziness. We thus sought to compare features of nystagmus at various stages following unilateral vestibular loss (UVL).

**Methods:**

We enrolled thirty consecutive patients after acute UVL with impaired vestibulo-ocular reflex (VOR) gain. The patients were allocated into three groups according to time from onset of symptoms: acute (1–7 days), subacute (8–30 days), and chronic (>30 days). Patients underwent video-oculography (with and without fixation) and video head impulse testing (vHIT) to determine VOR gain. We examined the relationships amongst SPV, VOR gain, and time from symptom onset across groups.

**Results:**

There were 11, 10, and 9 patients in the acute, subacute, and chronic stages of UVL, respectively. With visual fixation, only 8 patients (26.7%) demonstrated nystagmus, all from the acute group. With fixation removed, 26 patients (86.7%) exhibited spontaneous nystagmus, including 90.9%, 90%, and 77.8% of the patients from the acute, subacute, and chronic groups, respectively. Horizontal nystagmus was paralytic (i.e., fast phase contralesional) in 25 (96.7%) cases. Horizontal SPV was negatively correlated with logarithm of time from onset to examination (*r* = −0.48, *p* = 0.007) and weakly negatively correlated with ipsilesional VOR gain (*r* = −0.325, *p* = 0.08).

**Conclusion:**

In the subacute or chronic stages of UVL, paralytic nystagmus with fixation removed persisted at a low intensity. Therefore, weak nystagmus in the dark may have diagnostic value in chronic dizziness.

## Introduction

Nystagmus is an involuntary “to and fro movement” of the eyes, which can be highly localizing in patients with acute dizziness and vertigo. One common cause of vertigo is acute unilateral vestibular loss (UVL), for example from vestibular neuritis. Unilateral vestibular loss (UVL) typically presents with horizontal-predominant nystagmus with the fast phase toward the contralesional side (1).

Vestibular recovery following unilateral vestibular loss is a dynamic process. The spontaneous nystagmus, which often starts strong, gradually gets weaker with time as vestibular compensation begins. One technique to enhance detection of subtle nystagmus at the bedside is to remove fixation (i.e., to remove the stationary visual signal that can suppress VOR and inhibit nystagmus). Conventional teaching is that removing visual fixation only enhances peripheral nystagmus (2). However, studies have also demonstrated that some central nystagmus can be enhanced by removing visual fixation (3, 4).

In clinical settings, various tools are used to remove visual fixation, such as the penlight cover test (5), occlusive ophthalmoscopy (6), optical Frenzel goggles, video-infrared goggles examination, and video-oculography (VOG). Among them, VOG systems that employ infrared goggles can completely remove visual fixation.

The clinical significance of weak nystagmus with removal of fixation (i.e., in the dark) remains unclear. Studies have demonstrated that some healthy individuals present with weak spontaneous or positional nystagmus in the dark (7, 8). This finding in normal subjects can make it difficult to interpret the relevance of low-intensity nystagmus, particularly those with a slow-phase velocity (SPV) of <3°/s (9).

To date, most studies have focused on ocular motor signs during the acute stage of vertigo (10, 11), rather than the subacute or chronic phases (12). In clinical practice, however, patients often present after the acute phase. We therefore need better data on nystagmus intensity in the subacute and chronic phases of peripheral loss (ideally, a clinical cut-point that can help determine whether a given weak nystagmus is likely to represent recovering vestibular loss versus a normal finding).

In the present study, we investigated the direction and intensity of nystagmus in patients who presented at differing time-points after (initially acute) UVL. We sought to determine (i) how commonly weak nystagmus persists in the dark after acute UVL and (ii) whether weak nystagmus has localizing value (i.e., can it predict the lesion side?).

## Method

We retrospectively reviewed consecutive patients who presented to our outpatient department for dizziness or vertigo between July 2019 and June 2021. All patients underwent structured histories, complete neurological and oto-neurological examinations, and video-infrared goggles examination (with skull vibration and head-shaking as provocative maneuvers for nystagmus). All underwent VOG (including quantitative nystagmus measurement) and video head impulse testing (vHIT) simultaneously, which was recorded using a VOG/vHIT system (EyeSeeCam; Middelfart, Demark). Brain MRI was performed when focal neurological signs or central vestibular signs were identified. The patients who experienced acute vestibular syndrome at onset of dizziness and had a VOR gain of <0.8 on one side during vHIT were included for subsequent analysis. Patients with central vestibulopathy confirmed by MRI (i.e., a structural lesion) were excluded from this study. Patients who took any vestibular suppressant in the 48 h prior to vestibular testing were excluded. Patients who underwent vestibular rehabilitation before VOG examination were similarly excluded. The present study was performed in accordance with the ethical standards set forth in the 1964 Declaration of Helsinki and its subsequent amendments, and it was approved by the Institutional Review Board of the Research Ethics Committee of Taichung Tzu Chi Hospital (REC109-64).

During VOG examination, spontaneous nystagmus with and without visual fixation was recorded when the patients sat upright with their heads in neutral position and straight-ahead gaze. The presence or absence of nystagmus was determined by a subspecialty-trained vestibular neurologist (TPC) on the basis of VOG traces and videos. When nystagmus was present, the SPVs of the horizontal and vertical components were calculated. The lesion side was determined through vHIT, where which the lesioned VOR gain was defined as <0.8. On the basis of the time from symptom onset to VOG and vHIT, the included patients’ UVL were classified as being in the acute (1–7 days), subacute (8–30 days), or chronic (>30 days) stage. We compared the SPVs of nystagmus across the acute, subacute, and chronic stages. We also measured the relationships (i) between SPV and VOR gain, (ii) between SPV and time from onset to examination, and (iii) between VOR gain and time from onset to examination.

Descriptive statistics were applied to the collected demographic data and the data on the prevalence of nystagmus at various stages of UVL. Mann–Whitney U test was performed to compare the SPVs between stage groups. Pearson’s correlation coefficients were calculated to determine the correlations between SPV, VOR gain, and the time from onset to examination.

## Results

Thirty patients with UVL were included in our study. Among the included patients, 18 (60%) were male, their mean age was 54.1 years (range, 32–76 years), 16 (53.3%) had right vestibular loss, and 14 had left vestibular loss. The etiologies comprised acute unilateral vestibulopathy/vestibular neuritis (*n* = 25), Ramsay Hunt syndrome (*n* = 3), and acute unilateral audio-vestibular loss (*n* = 2). None of the included patients had middle ear symptoms or prior history of middle ear diseases.

On the basis of the timing of VOG and vHIT examinations relative to symptom onset, 11, 10, and 9 patients were determined to be in the acute stage (1–7 days), subacute stage (8–30 days), and chronic stage (>30 days) of UVL, respectively. The mean time from onset of vestibular syndrome to exam was 4.1 days in acute stage, 21.1 days in subacute stage, and 192.3 days in chronic stage (range 2–409 days). All included patients were symptomatic (i.e., had dizziness or vertigo) at the time of examination. With visual fixation, 8 patients (72.7%) in the acute stage had spontaneous nystagmus (mean horizontal SPV, 0.76°/s), whereas none in the subacute or chronic stage presented with spontaneous nystagmus.

**Figure 1:**
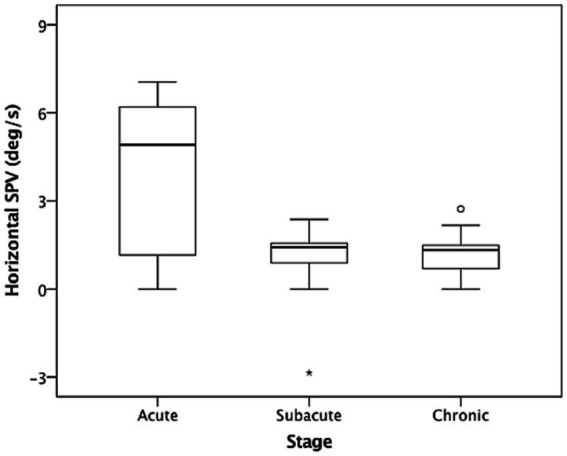
Nystagmus slow phase velocities (SPVs) in the dark in acute stage (1–7 days), subacute stage (8–30 days), and chronic stage (>30 days) after acute unilateral vestibular loss. The box and whisker plots present median values (central line), upper and lower quartiles, upper and lower extremes, and outliers.

**Figure 2:**
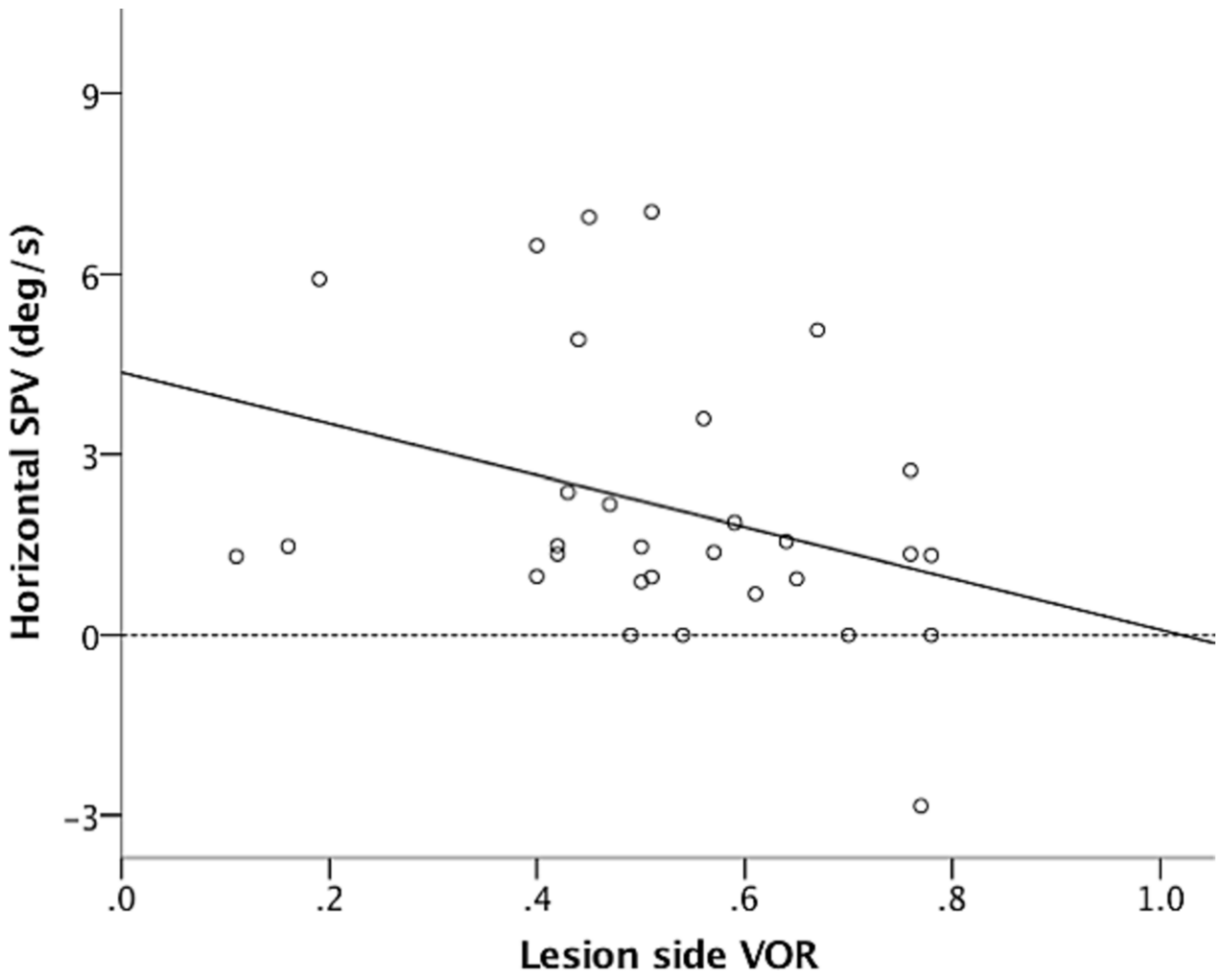
Horizontal slow-phase velocities (SPV) tended to be negatively correlated with ipsilesional VOR gain (*r* = −0.325, *p* = 0.08). Solid and dotted lines are regression and zero-horizontal lines, respectively.

**Figure 3:**
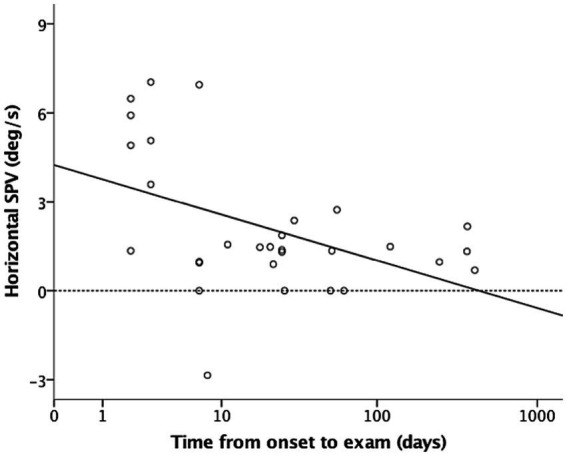
Nystagmus weakened rapidly after acute stage unilateral vestibular loss and was maintained at a low intensity (0.69°/s and 2.73°/s). Horizontal slow-phase velocity (SPV) was negatively correlated with logarithm of time from onset to examination (*r* = −0.48, *p* = 0.007). *X*-axis scale is logarithmic. Solid and dotted lines are regression and zero-horizontal lines.

In the dark (i.e., without fixation), 26 of the included patients (86.7%) had spontaneous nystagmus, including 90.9%, 90%, and 77.8% of the patients in the acute stage, subacute stage, and chronic stage, respectively. Among the included patients, 25 (96.7%) presented with paralytic nystagmus, in which the fast phase was directed toward the contralesional side. One patient displayed recovery nystagmus in which the fast phase was directed toward the ipsilesional side. Quantitative analysis revealed that the mean horizontal SPVs were 3.93°/s, 0.95°/s, and 1.19°/s in the acute stage, subacute stage, and chronic stage, respectively. The distribution is presented in Figure 1. The SPV tended to be higher in the acute stage (acute vs. subacute, *p* = 0.06; acute vs. chronic, *p* = 0.04, Mann–Whitney U test), but not different between subacute and chronic stages (*p* = 0.84, Mann–Whitney U test). The horizontal SPV was negatively correlated with the ipsilesional VOR gain but not to a significant level (*r* = −0.325, *p* = 0.08; Figure 2). The horizontal SPV was not correlated with the time from onset to examination (*r* = −0.202, *p* = 0.28). However, it was negatively correlated with the logarithm of time (*r* = −0.48, *p* = 0.007). Most SPV values recorded in the first week (63.6%) were greater than 3°/s. Thereafter, the recorded SPVs decreased sharply. However, most of the nystagmus in the subacute and chronic stages persisted at a low intensity (SPV, 0.69°/s–2.73°/s). Three patients still had nystagmus 1 year after vertigo onset. Weak paralytic nystagmus (SPV, 0.69°/s) was observed for up to 409 days (Figure 3) in one case.

Of the 30 included patients, 10 (33.3%) also had vertical components to their nystagmus. All vertical components were upbeating (vertical SPV, 0.73°/s–5.66°/s), and 70% of the upbeat-component nystagmus cases were in patients seen in the acute stage. Among the patients with provoked nystagmus, 28 had skull vibration–induced nystagmus, and 27 had head-shaking-induced nystagmus. All provoked nystagmus was paralytic (i.e., with fast phase directed toward the contralesional side).

Only one patient with left vestibular neuritis had recovery nystagmus, which was observed 8 days after vertigo onset. This spontaneous nystagmus was left-beating. By contrast, the patient’s skull vibration–induced and head-shaking nystagmus were right-beating.

## Discussion

In the present study, we investigated nystagmus characteristics in patients who were at different stages following onset of unilateral vestibular loss. Nystagmus with fixation disappeared beyond 1 week of symptom onset of vestibular loss. By contrast, most patients continued to demonstrate weak nystagmus (SPV < 3°/s) in the absence of fixation (in other words, in the dark) for months. Notably, this faint nystagmus was still observed in 3 patients who were examined more than 1 year after onset. The longest interval was 409 days. Of the 26 cases with nystagmus, 25 exhibited nystagmus beating away from the lesion side, indicating the presence of paralytic nystagmus. Several patients (36.4%) in the acute stage only exhibited weak nystagmus (SPV < 3°/s) even when examinations were conducted in the absence of fixation.

A number of compensatory mechanisms begin soon after a vestibular insult and may be attributed to either static vestibular compensation or vestibular restoration (i.e., recovery of vestibular hair cells or the vestibular nerve) (13). These processes cause spontaneous nystagmus to decay over time following acute vestibular injury. Rodents can achieve complete static vestibular compensation in 1 week (14), and cats can complete this process in 6 weeks (15, 16). By contrast, the time required to complete static compensation varies widely across human individuals. An early study by Matsuzaki et al. indicated that the spontaneous nystagmus of most people completely disappeared within 1 month after vestibular neuritis (17). However, Matsuzaki used optical Frenzel goggles, which could not completely remove visual fixation. In a study that used infrared goggles to block fixation, only 2 of 20 patients exhibited weak spontaneous nystagmus at 3 months after symptom onset, and only 1 still had nystagmus 1 year after onset (12). However, 35% of patients in that study had reversal of their canal paresis by calorics, suggesting that a part of their nystagmus disappeared because of vestibular restoration. Another long-term follow-up study reexamined 18 patients 7–8 years after the onset of vestibular neuritis, and it reported that 7 (38.9%) patients still displayed weak spontaneous nystagmus, which was identified through electro-oculography (18). Caloric testing revealed that among the 18 patients, 10 (55.6%) exhibited vestibular restoration and eight did not. In contrast to the aforementioned studies, the patients included in our study all exhibited incomplete vestibular restoration (as indicated by their vHIT results) at the time of enrollment. Consequently, 25 (83.3%) of the included patients presented with paralytic nystagmus and only one patient had recovery nystagmus. Our study demonstrated that when vestibular restoration is incomplete, static vestibular compensation is usually insufficient to completely overcome imbalances, resulting in residual weak nystagmus in the dark. This weak spontaneous nystagmus reflects the patients’ inability to establish stable static compensation and is associated with the chronic dizziness of these patients. Interestingly, there may have been a plateau of static compensation in these patients since the SPV was similar in subacute and chronic patients (in other words, nystagmus did not weaken further beyond the subacute phase). A prospective, longitudinal study with repeated measurement of SPV in the same patients is required to examine the existence of this plateau.

With the development of video oculomotor recording systems, infrared goggles can be effectively used to detect weak nystagmus. However, the clinical relevance of weak nystagmus is still being debated. In a study, portable VOG with fixation blocking revealed a high prevalence (30.7%) of low-velocity spontaneous nystagmus in 100 healthy individuals (7). This finding also corresponds to those of several other studies, which detected weak nystagmus (SPV, 0.7°/s–5.0°/s) in some healthy individuals (8, 19, 20). Collectively, these literature findings contribute to the ongoing debate about the clinical relevance of weak nystagmus. The present study showed that most patients in the subacute and chronic stages following unilateral vestibular loss had persisting low-intensity nystagmus that could only be detected with fixation removal. Another similar study examined 22 patients 2 months after acute vestibular neuritis and found that ten manifested weak nystagmus (SPV, 0°/s–3°/s) (21). If weak nystagmus is clinically nonsignificant, the directions of nystagmus should be evenly distributed between the ipsilesional and contralesional sides. Our study indicated that 96.2% of the nystagmus cases beat toward the contralesional side (i.e., were paralytic nystagmus), demonstrating that weak nystagmus in UVL is likely clinically significant and an indicator of the lesion side. There is likely an overlap in the range of intensity of nystagmus between physiologic nystagmus and the weak, but clinically significant, nystagmus seen in the subacute and chronic stages following unilateral loss. Thus, for patients with the correct history (onset of dizziness weeks to months prior), weak nystagmus should be carefully examined for, including with removal of visual fixation. For clinical settings without specialized equipment (e.g., infrared video-oculography or optical Frenzels), fixation can be easily removed at the bedside by means of the penlight-cover test, or by occlusive ophthalmoscopy (5, 6). Because of the overlap in nystagmus intensity, it is hard to distinguish weak pathological nystagmus from physiologic nystagmus simply using an SPV cut-off. Instead, the presence and intensity of nystagmus should be considered within the broader clinical context (clinical history, vestibular findings, ocular motor signs). Together, these features help identify when weak nystagmus may be pathologic and/or provide useful diagnostic information. If its clinical relevance remains undetermined, the weak nystagmus should be closely followed up to observe how the nystagmus changes over time.

Our study has several limitations. First, it was not a longitudinal study, and included patients were not individually followed up at multiple time points to monitor changes in nystagmus. Second, our study was a retrospective study with a small sample size. Prospective studies with larger sample sizes are needed to confirm our findings.

## Conclusion

Nystagmus with visual fixation may begin to disappear by as soon as 1 week after onset of acute unilateral vestibular loss. By contrast, nystagmus in the dark persisted at a low intensity for months, and in most cases the direction was consistent with the expected paralytic nystagmus (i.e., contralesional). Therefore, although subtle nystagmus in the dark is not always pathological, it can be of clinical significance in patients with chronic dizziness and should not be overlooked.

## Data availability statement

The raw data supporting the conclusions of this article will be made available by the authors, without undue reservation.

## Ethics statement

The studies involving humans were approved by the Institutional Review Board of the Research Ethics Committee of Taichung Tzu Chi Hospital (REC109-64). The studies were conducted in accordance with the local legislation and institutional requirements. Written informed consent for participation was not required from the participants or the participants' legal guardians/next of kin because this is a retrospective study.

## Author contributions

C-CC: Data curation, Formal analysis, Methodology, Writing – original draft. AB: Writing – review & editing. T-PC: Conceptualization, Data curation, Formal analysis, Funding acquisition, Methodology, Supervision, Writing – original draft, Writing – review & editing.
